# HYSOGs250m, global gridded hydrologic soil groups for curve-number-based runoff modeling

**DOI:** 10.1038/sdata.2018.91

**Published:** 2018-05-15

**Authors:** C. Wade Ross, Lara Prihodko, Julius Anchang, Sanath Kumar, Wenjie Ji, Niall P. Hanan

**Affiliations:** 1 New Mexico State University, Department of Plant and Environmental Sciences, Las Cruces, New Mexico 88003, USA; 2 New Mexico State University, Department of Animal and Range Sciences, Las Cruces, New Mexico 88003, USA

**Keywords:** Hydrology, Environmental chemistry

## Abstract

Hydrologic soil groups (HSGs) are a fundamental component of the USDA curve-number (CN) method for estimation of rainfall runoff; yet these data are not readily available in a format or spatial-resolution suitable for regional- and global-scale modeling applications. We developed a globally consistent, gridded dataset defining HSGs from soil texture, bedrock depth, and groundwater. The resulting data product—HYSOGs250m—represents runoff potential at 250 m spatial resolution. Our analysis indicates that the global distribution of soil is dominated by moderately high runoff potential, followed by moderately low, high, and low runoff potential. Low runoff potential, sandy soils are found primarily in parts of the Sahara and Arabian Deserts. High runoff potential soils occur predominantly within tropical and sub-tropical regions. No clear pattern could be discerned for moderately low runoff potential soils, as they occur in arid and humid environments and at both high and low elevations. Potential applications of this data include CN-based runoff modeling, flood risk assessment, and as a covariate for biogeographical analysis of vegetation distributions.

## Background & Summary

Soils have a fundamental role in the global hydrologic cycle by governing rainfall infiltration and groundwater recharge, which ultimately affects the lateral transport of water and subsequent runoff potential. Knowledge of soil hydraulic properties is therefore of interest to ecologists, hydrologists, and soil scientists, and is critical for parameterization of a variety of empirical and physically-based hydrologic models, dynamic-vegetation models, and land-surface models^1–3^.

The U.S. Department of Agriculture (USDA) curve-number (CN) method provides a simplified approach to the estimation of key hydrologic processes while being grounded in a physical understanding of saturated flow and runoff processes^4–6^. The CN method avoids the problems inherent to parameterizing and running more complex models due to its simplicity and relatively low data input requirements, and has been implemented in a variety of hydrologic, erosion, and water-quality models^7–9^. CN selection is derived from the hydrologic response of various combinations of soil types and land cover classes^2,10^. Particularly relevant to the subject of this analysis, and the data product we make available, is the classification and development of soil parameters for CN-based runoff modeling. The lack of globally consistent data derived from contemporary soil information served as the overarching motivation for this analysis.

CN-based runoff estimates require information regarding the minimum infiltration rate of rainfall into the soil and the transmission rate of groundwater through the soil profile after prolonged wetting. Runoff occurs when the rainfall rate exceeds the infiltration capacity of soils. The rate at which these processes occur is primarily affected by the physical nature of soils (e.g., texture, compaction), in addition to land cover, antecedent moisture, and rainfall intensity. For example, coarse-textured sandy soils have larger pore spacing, allowing water to infiltrate quickly relative to fine-textured clay soils.

Soils are thus classified into four hydrologic soil groups (HSGs) to infer runoff potential (Table 1)^11^. HSG-A has the lowest runoff potential (typically contains more than 90% sand and less than 10% clay), HSG-B has moderately low runoff potential (typically contains between 10 to 20% clay and 50 to 90% sand), HSG-C has moderately high runoff potential (typically contains between 20 to 40% clay and less than 50% sand), and HSG-D has high runoff potential (typically contains more than 40% clay and less than 50% sand). Classification is determined by the least transmissive soil layer—often measured as saturated hydraulic conductivity (K_s_)—depth to water table or depth to an impermeable layer (e.g., duripan, bedrock). If K_s_ is unknown or not available, infiltration and transmission rates can be inferred from soil texture, with the underlying assumption that soils with similar content of sand, silt, and clay have analogous hydraulic properties^12–14^. Wet soils have high runoff potential (regardless of texture) due to the presence of a groundwater table within 60 cm of the surface. These soils are assigned dual HSGs, as a less restrictive group can be assigned (according to texture or K_S_) if they can be adequately drained.

**Figure 1:**
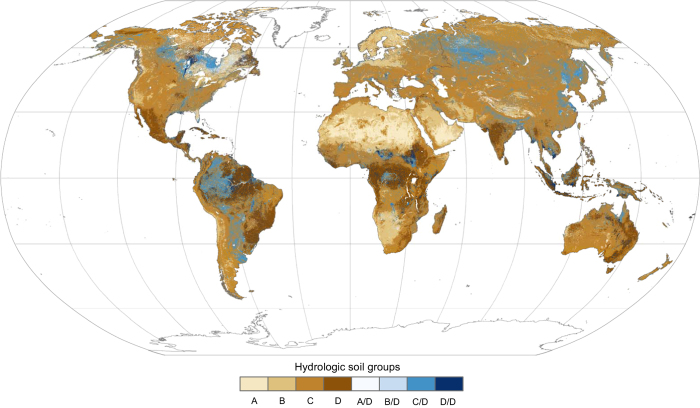
Global distribution of hydrologic soil groups. Hydrologic soil groups A, B, C, and D correspond to low, moderately low, moderately high, and high runoff potential, respectively. Wet soils are assigned a dual HSG (e.g., HSG A/D) and have high runoff potential due to the presence of a water table within 60 cm of the surface. A less restrictive group can be assigned if these soils are drained (e.g., HSG-A).

We derived HSGs from texture classes in accordance with USDA^11^ specifications (Table 1). The resulting data product—HYSOGs250m—represents typical soil runoff potential suitable for regional, continental, and global scale analyses and is available in a gridded format at a spatial resolution of 250 m (Fig. 1).

Our analysis indicates that soils with moderately high runoff potential dominate the global distribution (57.4%), followed by soils with moderately low (HSG-B 12.2%), high (HSG-D 10.1%), and low runoff potential (HSG-A 3.0%) (Table 2). Dual HSGs A/D, B/D, C/D, and D/D accounted for 0, 1.4, 13.5, and 2.4% of the global distribution, respectively. Some global trends were observed for soils with high and low runoff potential. Low runoff potential soils are found predominantly in parts of the Sahara and Arabian Deserts, which are characterized by very deep and well-drained sandy soils. High runoff potential soils occur predominantly within tropical and sub-tropical zones (with notable additions occurring in the Alaska-Yukon Arctic and Canadian Taiga and Boreal Shield) and are characterized by soils with high clay content or shallow soils (<50 cm to bedrock). No clear pattern could be discerned for soils with moderately low runoff potential at the global scale, as these HSGs occur in arid and humid environments and at both high and low elevations.

## Methods

**Figure 2:**
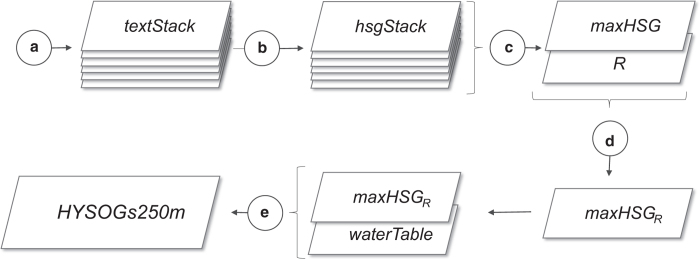
Conceptual framework illustrating the workflow used to develop HYSOGs250m. textStack represents USDA-based soilGrids250m texture classes^15^ for six depth intervals (0, 5, 15, 30, 60, and 100 cm); hsgStack represents hydrologic soil group (HSGs) classified from each texture class, maxHSG represents HSGs defined by the most restrictive layer (0 to 1 m), R represents bedrock depth^15^, maxHSG_R_ represents HSGs re-classified to the bedrock depth criteria, and waterTable represents the HSGs reclassified to account for both the depth to bedrock and the water table criteria.

**Figure 3:**
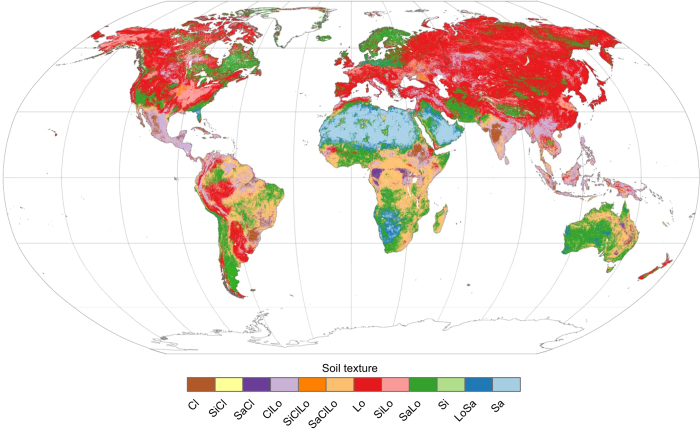
USDA-based soil texture classes. Adapted from the Food and Agriculture (FAO)^15^. Cl is clay, SiCl is silty clay, SaCl is sandy clay, ClLo is clay loam, SiClLo is silty clay loam, SaClLo is sandy clay loam, Lo is loam, SiLo is silty loam, SaLo is sandy loam, Si is silt, LoSa is loamy sand, Sa is sand. Note that mapped texture classes represent the soil surface (0 cm).

**Figure 4:**
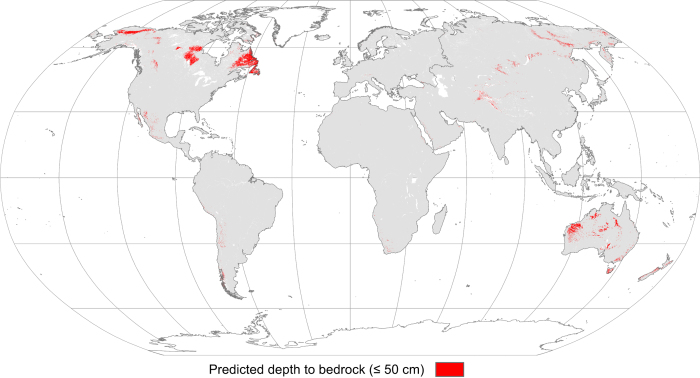
Predicted depth to bedrock within 50 cm of the surface. Adapted from the Food and Agriculture (FAO)^15^. Note that individual grid cells (bedrock occurrence) may not be visible at the global scale.

**Figure 5:**
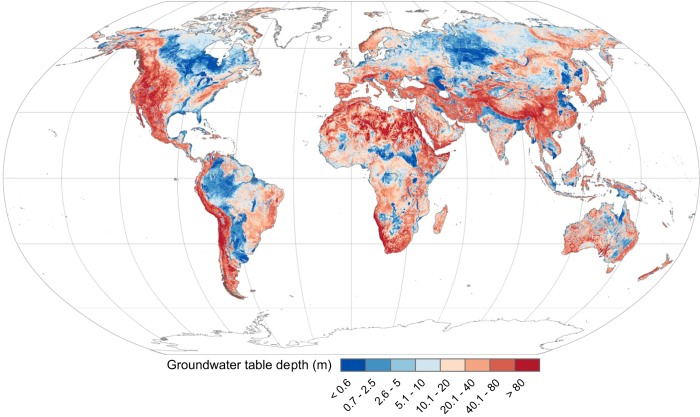
Groundwater table depth. Adapted from Fan *et al*.^16^ Dual hydrologic soil groups were assigned to grid cells (pedons) based upon the presence of a water table (<60 cm of the surface).

The process for producing HYSOGs250m consisted of five primary steps (Fig. 2). We classified HSGs from USDA-based soil texture classes (Fig. 3), depth to bedrock (Fig. 4), and groundwater table depth (Fig. 5) as specified by the USDA-Natural Resources Conservation Service (USDA-NRCS) National Engineering Handbook (NEH)^11^. Soil texture classes and depth to bedrock were obtained from the United Nations (UN) Food and Agriculture Organization (FAO) soilGrids250m system^15^. These data and associated meta-data are available for download as GeoTiffs at ftp://ftp.soilgrids.org/data/recent. Groundwater table depth^16^ and associated meta-data are available for download as NetCDF at https://glowasis.deltares.nl/thredds/catalog/opendap/opendap/Equilibrium_Water_Table/catalog.html. All computations were performed within the R open source environment for statistical computing^17^ and functions from the raster package^18^.

Soil texture to 1 m depth was represented with FAO soilGrids250m texture classes at six depths: 0, 5, 15, 30, 60, and 100 cm. The soilGrids were stacked into a multi-band raster (textStack) using the raster::stack function (Fig. 2a). For the purpose of this analysis, we refer to individual grid cells (~250 m×250 m) in the raster stack (1 m depth) as soil pedons. Each grid cell in the raster stack (or pedon) was re-classified into one of four HSGs (hsgStack) using the classification scheme reported in Table 1 (Fig. 2b). This allowed us to infer the water transmissivity of each layer in the profile from the stacked texture classes. Note that integers 1, 2, 3, and 4 were used to represent HSGs A, B, C, and D, respectively. The raster::max function (Fig. 2c) was then used to determine the largest value of each grid cell in the raster stack, allowing us to infer the most restrictive layer in the pedon. This value (maxHSG) was used to assign HSGs for each pixel in the stack, thus representing soil runoff potential for each pedon. Shallow soils (bedrock within 50 cm of the surface, Fig. 4) were re-classified to HSG-D (maxHSG_R_, Fig. 2d). Dual HSGs were assigned to pedons with shallow water tables (<60 cm from the surface) using the depth to groundwater table dataset^16^ (Fig. 2e). Integers 11, 12, 13, and 14 were used to denote dual HSGs A/D, B/D, C/D, and D/D, respectively.

### Code availability

The R code used to develop HYSOGs250m, described in Fig. 2, is available for download from the Oak Ridge National Laboratory (ORNL) Distributed Active Archive Center (DAAC) (Data Citation 1).

## Data Records

HYSOGs250m (Data Citation 1) is available for download as an un-projected GeoTiff at 7.5 arc-second (approximately 250 m resolution). The value column variables 1, 2, 3, 4, 11, 12, 13, and 14 correspond to HSG A, B, C, D, A/D B/D, C/D, and D/D, respectively.

## Technical Validation

We briefly describe uncertainty assessments of the FAO soilGids^15^ and groundwater table depth^16^ data that were used as input for our analysis; however, readers are referred to the corresponding publications for a detailed description of the methods and uncertainty analysis.

### SoilGrids

Soil profile data was compiled by the FAO from approximately 150,000 unique sites covering every continent; however, the tropics, semi-arid to hyper-arid regions, and mountain regions were underrepresented^15^. Furthermore, soils with high runoff potential are likely under-estimated due to the uncertainty associated with depth to bedrock^15^. However, their depth to bedrock models performed reasonably well, and explained more than 50% of the global variation (R^2^=0.54).

Accuracy assessment was performed with 10-fold repeated cross-validation using soil profile data from ca. 150 000 globally distributed sites used to develop soilGrids250m^15^. In all instances, the amount of variation explained by the soil texture models was higher than 72.6%; root mean square error (RMSE) was lowest for clay (9.5%), followed by silt (9.8%), and sand (13.1%)^15^.

### Groundwater table depth

A total of 1,603,781 well sites were compiled from government archives and published literature to generate predictions of global groundwater table depth^16^. On average, the modeled groundwater table was 1.62 m (±17.91 m) lower than observations at the global scale. Note that local, perched aquifers were not modeled^16^. Groundwater pumping, drainage, and irrigation were not represented, thus neglecting the local complexity of human influence and only capturing the broad-scale patterns of groundwater^16^.

### Comparison with other datasets

Hong and Adler^19^ reported that the global distribution of soils was dominated by moderately low runoff potential (36.8%), followed by high (25.3%), low (20.5%), and moderately high (17.4%) runoff potential. Although this is in stark contrast with what we report, these discrepancies are largely attributed to different classification schemes (Table 1), and to a lesser extent, different methodologies.

For comparative purposes only, we used the same classification scheme reported by Hong and Adler^12,19^. This comparison revealed that the distribution of the two datasets were in closer agreement, and that soils are dominated by moderately low runoff potential (37%), followed by high (32%), low (17%), and moderately high (15%) runoff potential. However, it is important to note that the classification scheme reported by Hong and Adler was based on earlier work by Musgrave^13^ using rainfall, runoff, and infiltrometer measurements^13^, a practice that has since been abandoned by the USDA^11^. Furthermore, the deprecated classification scheme does not account for the presence of impermeable layers (e.g., bedrock) or depth to groundwater table.

### Other considerations

**Figure 6:**
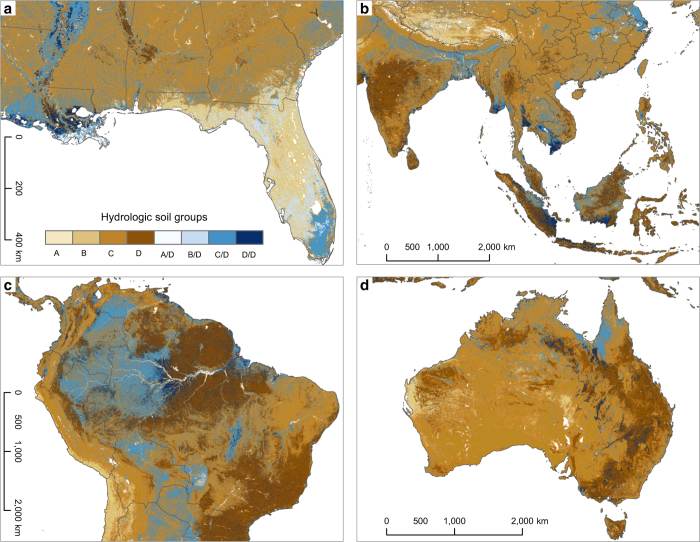
Distribution of hydrologic soil groups for select regions. (**a**) Southeast US, (**b**) Southeast Asia, (**c**) northern South America, (**d**) Australia.

Note that substantial variation can exist within and between soil texture classes and their respective hydraulic properties (Fig. 6). According to the revised NEH^11^, HSG-A *typically* consists soils classified as sand (e.g, more than 90% sand and less than 10% clay content), but can include loamy sand, sandy loam, loam, or silt loam. Likewise, HSG-B typically consists of loamy sand and sandy loam, but can contain loam, silt loam, silt, or sandy clay loam, while HSG-C typically consists of loam, silt loam, sandy clay loam, clay loam, and silty clay loam, but can include clay, silty clay, and sandy clay textures^11^.

## Usage Notes

Users of this dataset should be aware that HYSOGs250m represents general patterns of soil runoff potential appropriate for regional- to global-scale analyses and may not capture the local variance suitable for fine-scale applications. Although originally developed to support CN-based computations of rainfall runoff, HYSOGs250m can be used as a covariate for empirical analyses investigating various soil-environmental relationships. For example, plant and/or animal species distributions are often related to soil texture, plant available water, and groundwater. HYSOGs250m may be a useful covariate to further explain such relationships, as these data were produced by incorporating depth to bedrock, depth to groundwater table, and soil texture classes. These data can also be used for flood risk assessment and suitability mapping. End-users who are not interested in dual HSGs may simply re-classify HSGs A/D, B/D, C/D, and D/D to HSG-D.

## Additional information

**How to cite this article:** Ross, C. W. *et al.* HYSOGs250m, global gridded hydrologic soil groups for curve-number-based runoff modeling. *Sci. Data* 5:180091 doi: 10.1038/sdata.2018.91 (2018).

**Publisher’s note:** Springer Nature remains neutral with regard to jurisdictional claims in published maps and institutional affiliations.

## Supplementary Material



## Figures and Tables

**Table 1:** Hydrologic soil groups (HSGs) classification scheme.

**HSG**	**Runoff potential**	**Texture class**	a**Hong and Adler**
A	Low	Sa	Sa, SaLo, LoSa
B	Moderately low	SaLo, LoSa	Si, Lo, SiLo
C	Moderately high	ClLo, SiClLo, SaClLo, Lo, SiLo, Si	SaClLo
D	High	Cl, SiCl, SaCl,	ClLo, SiClLo, SaCl, SiCl, Cl
USDA texture classes, where Sa is sand, SaLo is sandy loam, LoSa is loamy sand, ClLo is clay loam, SiClLo is silty clay loam, SaClLo is sandy clay loam, Lo is loam, SiLo is silty loam, Si is silt, Cl is clay, SiCl is silty clay, SaCl is sandy clay.			

^a^Soil texture classes used for HSG assignment reported by Hong and Adler^19^.

**Table 2:** Global and continental distribution of hydrologic soil groups (HSGs).

	**HSG coverage (%)**							
**Coverage**	**A**	**B**	**C**	**D**	**A/D**	**B/D**	**C/D**	**D/D**
Global	3.0	12.2	57.4	10.1	0.0	1.4	13.5	2.4
Africa	13.8	24.5	35.3	16.5	0.1	0.9	5.3	3.7
Asia	1.3	7.6	67.1	4.5	0.0	0.7	17.5	1.2
Australia	0.0	3.9	65.2	21.7	0.0	0.2	5.9	3.2
Europe	0.1	22.4	61.9	0.7	0.0	2.6	12.3	0.1
North America	0.0	11.4	60.8	9.0	0.0	3.4	13.2	2.1
South America	0.0	1.9	58.0	20.6	0.0	0.3	12.3	6.9
Oceania	0.0	3.9	46.9	23.3	0.0	0.2	18.9	6.7
