# Author Correction: HYSOGs250m, global gridded hydrologic soil groups for curve-number-based runoff modeling

**DOI:** 10.1038/s41597-019-0008-7

**Published:** 2019-02-26

**Authors:** C. Wade Ross, Lara Prihodko, Julius Anchang, Sanath Kumar, Wenjie Ji, Niall P. Hanan

**Affiliations:** 1 New Mexico State University, Department of Plant and Environmental Sciences, Las Cruces, New Mexico 88003 USA; 2 New Mexico State University, Department of Animal and Range Sciences, Las Cruces, New Mexico 88003 USA

Correction to: *Scientific Data* 10.1038/sdata.2018.91; published online 15 May 2018

The original version of this Data Descriptor incorrectly referenced the “United Nations (UN) Food and Agriculture Organization (FAO) soilGrids250m system”. This has been corrected to “SoilGrids predictions” throughout the text in both the HTML and PDF versions.

In addition, the legend of Figure 4 incorrectly cited reference 15 in the paper. This has been corrected in the HTML and PDF versions to:

20. Shangguan, W., Hengl, T., Jesus, J.M. de, Yuan, H., & Dai, Y. Mapping the global depth to bedrock for land surface modeling. *Journal of Advances in Modeling Earth Systems.* **9**, 65–88 (2017).

